# Contemporary frameless intracranial biopsy techniques: Might variation in safety and efficacy be expected?

**DOI:** 10.1007/s00701-015-2543-0

**Published:** 2015-08-29

**Authors:** Iris S. C. Verploegh, Victor Volovici, Iain K. Haitsma, Joost W. Schouten, Clemens M. Dirven, Johan M. Kros, Ruben Dammers

**Affiliations:** Department of Neurosurgery, Brain Tumor Center, Erasmus Medical Center, ‘s Gravendijkwal 230, Kamer H-703, 3015 CE Rotterdam, The Netherlands; Department of Pathology, Brain Tumor Center, Erasmus Medical Center, Rotterdam, The Netherlands

**Keywords:** Frameless stereotaxy, Biopsy, Varioguide, Stealth, Brain tumor

## Abstract

**Background:**

Frameless stereotactic neuronavigation has proven to be a feasible technology to acquire brain biopsies with good accuracy and little morbidity and mortality. New systems are constantly introduced into the neurosurgical armamentarium, although few studies have actually evaluated and compared the diagnostic yield, morbidity, and mortality of various manufacturer’s frameless neuronavigation systems. The present study reports our experience with brain biopsy procedures performed using both the Medtronic Stealth Treon^TM^ Vertek® and BrainLAB® Varioguide frameless stereotactic brain biopsy systems.

**Patients and methods:**

All 247 consecutive biopsies from January 2008 until May 2013 were evaluated retrospectively. One hundred two biopsies each were performed using the Medtronic (2008–2009) and BrainLAB® system (2011–2013), respectively. The year 2010 was considered a transition year, in which 43 biopsies were performed with either system. Patient demographics, perioperative characteristics, and histological diagnosis were reviewed, and a comparison was made between the two brain biopsy systems.

**Results:**

The overall diagnostic yield was 94.6 %, i.e., 11 biopsies were nondiagnostic, 5 (4.9 %) with the Medtronic and 6 (5.9 %) with the BrainLAB® system. No differences besides the operating time (108 *vs* 120 min) were found between the two biopsy methods. On average, 6.6 tissue samples were taken with either technique. Peri- and postoperative complications were seen in 5.3 % and 12.9 %, consisting of three symptomatic hemorrhages (1.2 %). Biopsy-related mortality occurred in 0.8 % of all biopsies.

**Conclusions:**

Regarding diagnostic yield, complication rate, and biopsy-related mortality, there seems to be no difference between the frameless biopsy technique from Medtronic and BrainLAB®. In contemporary time, the neurosurgeon has many tools to choose from, all with a relatively fast learning curve and ever improving feasibility. Thus, the issue of choice involves not the results, but the familiarity, end-user friendliness, and overall comfort when operating the system.

## Introduction

The treatment of various intracranial lesions, despite progress in radiological techniques, largely still depends on the histological diagnosis. When open surgical biopsy or resection is not considered appropriate, brain needle biopsy has proven to be a safe and effective means of acquiring a diagnosis. Diagnostic yield has been shown to be between 83.6 and 100 %, with morbidity and mortality ranging from 0.7 to 16.1 % and 0 to 3.9 %, respectively [4]. Nevertheless, a question remains about the diagnostic accuracy, ranging 73 to 97 %, which might be due to sampling error and a lack of standardized criteria of diagnostic yield [4, 9]. Therefore, many techniques are currently employed to increase the yield as well as accuracy to improve the clinical significance and prognostic value of brain biopsy methods, such as radiological imaging techniques, intraoperative imaging, frozen section analysis, and even robotics [1, 2, 4, 8, 10–14, 16, 17].

There is no question that frameless stereotactic neuronavigation has been successfully proven to be a feasible technology to acquire brain biopsies with good accuracy and little mortality [4, 8, 11, 16]. Furthermore, its use fans out into others areas, such as placement of deep brain electrodes for epilepsy surgery or the treatment of Parkinson’s disease [5, 6]. Manufacturers of neuronavigation devices are rapidly introducing new frameless stereotaxy systems into the neurosurgical armamentarium. There are, however, no studies of which we are aware that have actually evaluated and compared the diagnostic yield, morbidity, and mortality of various manufacturer’s frameless neuronavigation systems in stereotactic frameless brain needle biopsies.

The present study reports our experience with brain biopsy procedures performed with both the Medtronic Stealth Treon^TM^ Vertek® and BrainLAB® Varioguide frameless stereotactic brain biopsy systems.

## Patients and methods

No IRB approval is needed for retrospective cohorts in academic centers in The Netherlands, so no informed consent was required from any of the patients. Files of all consecutive patients undergoing frameless image-guided stereotactic brain biopsy procedures at the Erasmus Medical Centre from January 2008 until and including May 2013 were reviewed. A total of 247 biopsies were performed in 242 patients. All biopsies performed before January 2010 were performed with the Medtronic Stealth Treon^TM^ Vertek® frameless stereotactic brain biopsy system. In this year the BrainLAB® Varioguide frameless stereotactic brain biopsy system was introduced in our clinic, which would replace the former. In the “transition” period between January and December 2010, all 43 biopsies were performed using one of the two frame-based techniques (19 with Medtronic Stealth Treon^TM^ Vertek® and 23 with Brainlab® Varioguide; for one patient the system was not reported in the operative report). Since January 2011 brain biopsies have been exclusively performed using the BrainLAB® Varioguide neuronavigation system. After the transition period, we had the Kolibri® system at our disposal, but we eventually switched to the Curve® BrainLAB® system, running these two in parallel in late 2012 and early 2013. The biopsy system itself, however, remained unchanged, viz. the Brainlab® Varioguide was used with both.

### Patient and preoperative neuroimaging characteristics

Age, gender, and biopsy-related complications (morbidity) and death (mortality) as well as operating time, biopsy method used, number of biopsies, and complications were extracted from case notes and operative reports. The anatomical site of the targeted lesion was obtained from preoperative computed tomography or magnetic resonance imaging scans that were acquired no more than 24 h preoperatively. The eventual diagnosis was obtained from the pathological report, prepared or supervised by JMK, from which the diagnostic yield was determined.

The issue of diagnostic yield is a delicate one, as detailed in several papers [2–4, 8, 9, 11]. Tumor biopsy is a process involving two potential sampling error points, the operation itself, depending on which area of the tumor the samples were taken from, and the pathological investigation, depending on what microscopic area of the acquired samples will be investigated. Therefore, we considered three possible diagnostic options, a full diagnosis, a partial diagnosis (pathologic tissue but no WHO classification possible), or no diagnosis at all. Because in the cases where the diagnosis was only partial a treatment could be initiated based on other characteristics of the specific patient (radiological aspect, speed of progression, oncological history), all biopsies where a treatment could be initiated and tumor tissue was obtained were considered diagnostic.

As such, a stereotactic biopsy serves its purpose if a specific patient can get the best specific treatment given all available data, and not only if it delivers a perfectly classifiable result, this sometimes being not possible because of shortcomings in the WHO classification.

The diagnostic yield of the biopsies was independently reviewed by two authors (RD and VV), and the results were discussed and validated to a common consensus.

### Operative technique

The techniques of frameless image-guided stereotactic biopsies have been described previously for the Stealth Treon^TM^ Vertek® system (Medtronic Inc., Minneapolis, MN, USA) [3] as well as the BrainLAB® Varioguide system (BrainLAB, Feldkirchen, Germany) [7, 19]. All biopsies were performed using the frameless stereotaxy protocol under general anesthesia and head fixation in a three-point Mayfield clamp. Intraoperative image guidance and the surgical plan were obtained using the Stealth Treon™ Vertek® system in 102 patients from January 2008 until December 2009 and with the BrainLAB® Varioguide system in 102 patients from January 2011 until May 2013, respectively. In the transition year 2010, 19 and 23 biopsies were performed with the Stealth Treon^TM^ Vertek® and BraiLAB® Varioguide system, respectively. For one patient, the biopsy system had not been reported in the operative report. As to the surgeon’s preference either fiducial markers and/or surface-merging techniques were used to enable operating room neuronavigational registration. The surgery was performed either by neurosurgeons or neurosurgical trainees under supervision of neurosurgeons. The trainees performing the surgery were split into two categories, junior residents, PGY (postgraduate years) 1–3, and senior residents, PGY 4–6. Tissue samples approximately 8 mm long and 1 mm thick were thus obtained.

In general, four biopsies were obtained at the preoperatively suggested target, as well as two to four more biopsies at a site proximal to the target on the same biopsy trajectory. The macroscopic appearance of the acquired tissue sample was assessed as described previously and, when appropriate, prompted to intraoperative freeze sectioning (10/247 = 4.0 %) [4]. If diagnostic tissue was not obtained, all parameters and measurements were rechecked to ensure accurate localization, after which a repeat biopsy was performed via the same or a new trajectory if needed.

### Statistical analysis

Database collection and statistical analysis were performed with SPSS 15.0 for Windows (SPSS Inc., Chicago, IL, USA). For comparison between the two frameless stereotaxy biopsy systems, continuous data were compared using one-way analysis of variance (ANOVA) testing for parametric data. For nonparametric data the Kruskal-Wallis one-way ANOVA test was used. These are presented as mean values ± standard deviations. For multiple comparisons the Bonferroni post-hoc correction was used. Proportions were compared with chi-square and Fisher’s exact test where appropriate and presented as percentages. P-values < 0.05 were considered statistically significant.

## Results

Table 1 summarizes the patient and perioperative characteristics with respect to the frameless stereotactic biopsy system used, i.e., Medtronic Treon^TM^ Vertek® *vs.* BrainLAB® Varioguide *vs.* the 2010 transition period. A higher proportion of males and a higher mean age was observed in the BrainLAB® group compared to the Medtronic Treon^TM^ group. The number of biopsies taken and perioperative complications were equal with both biopsy systems. Remarkably, the total operating time, including anesthetic preparation, was significantly longer using the BrainLAB® Varioguide.

**Table 1:** Patient and perioperative characteristics related to biopsy method

	Stealth Treon^TM^ (n = 102)	Transition period (n = 43)	BrainLAB® (n = 102)
Male (%)	49.0*	62.8	65.7
Age (years ± SD)	54.0 ± 19.7*	56.3 ± 20.0	60.1 ± 15.3
Operating time (min ± SD)	108 ± 36*	121 ± 26	120 ± 28
Number of biopsies ± SD	7.0 ± 2.9	6.6 ± 2.9	6.4 ± 2.8
Complication (%)	4.9	4.7	5.9
Hemorrhage	2.9	2.3	4.9
Technical failure	2.0	2.3	1.0

Shown are percentages and mean values ± standard deviations (SD). *p < 0.05

The distribution of the different tumor sites related to the biopsy system used is shown in Table 2. There were no statistically relevant differences between the system used for frameless brain biopsy and the various tumor locations.

**Table 2:** Preoperative anatomical localization of the intended lesion chosen for biopsy

	Stealth Treon^TM^(n = 102)	Transition period (n = 43)	BrainLAB® (n = 102)
Frontal	23	10	21
Temporal	11	5	16
Parietal	21	3	11
Occipital	5	1	4
Frontotemporal^*^	2	1	3
Frontoparietal	5	3	7
Parietooccipital	7	0	9
Parietotemporal	7	3	4
Temporooccipital	0	0	2
Thalamus/basal ganglia^*^	16	12	18
Pons/medulla oblongata cerebellopontine angle	1	3	2
Cerebellum	0	0	2
Supra-/intrasellar^**^	3	2	2
Third ventricle	0	0	1

### Length of operating time

Over the years that the BrainLAB® system was used (2010–2013) there was a gradual but nonsignificant decrease in operating time from a median of 130 min to 115 min in 2010 and 2013, respectively. Nevertheless, total operating time remained significantly longer for the BrainLAB® system. Correction for age, tumor site, or surgeon experience level (i.e., PGY 1–3, PGY 4–6, or neurosurgeon) did not change this finding. Therefore, it seems that a biopsy procedure with the BrainLAB® Varioguide on average takes some 10 min longer than a biopsy with the Medtronic Treon^TM^ Vertek®.

### Complications and mortality

Postoperative complications were encountered in 32 procedures (12.9 %), summarized in Table 3. Of these, three symptomatic hemorrhages (1.2 %) were diagnosed via postoperative computed tomography scanning. Furthermore, symptomatic postoperative edema was present in 6 (2.4 %) patients and early focal neurologic deterioration after biopsy was seen in 15 (6.1 %), of which 9 were observed in the BrainLAB® Varioguide group (8.8 %, p > 0.05, compared to the Medtronic Treon^TM^ group). The incidence of postoperative symptomatic hemorrhage between the two techniques was equal.

**Table 3:** Postoperative complications

(%)	Stealth Treon^TM^(n = 102)	Transition period (n = 43)	BrainLAB® (n = 102)
None	91.2	83.7	84.3
Hemorrhage	1.0	0	2.0
Edema formation	3.9	2.3	1.0
Focal neurology	2.9	7.0	8.8
Others^*^	1.0	4.7	3.9
Biopsy-related death	0	0	2.0

Data are presented as percentages. There are no differences between the biopsy procedures. ^*^Others include psychosis, hyperglycemia, epilepsy, hypertension, and diabetes insipidus

Biopsy-related death was defined as death occurring within 30 days as a result of symptomatic postoperative hemorrhage or edema formation and was observed in two patients, i.e., 0.8 %. There were no differences between the two biopsy systems.

### Diagnostic yield

The overall diagnostic yield was 94.6 %, i.e., a histological diagnosis was made in 193 of 204 patients or in 11 cases (5.4 %) the biopsy was nondiagnostic. This was not statistically different between the Medtronic Treon^TM^ (4.9 %) and BrainLAB® (5.9 %) stereotactic biopsy techniques. When including the transition period patients, the overall diagnostic yield was 93.5 % (231/247). A nondiagnostic biopsy was observed in 11.6 % in the transition period (p > 0.05). The lesion locations of nondiagnostic biopsies were frontal (n = 2), frontotemporal (n = 1), parietooccipital (n = 2), and in the third ventricle (n = 1) for BrainLAB®; parietal (n = 2), occipital (n = 1), parietooccipital (n = 1), and temporal (n = 1) for Medtronic Treon^TM^; frontoparietal (n = 1), temporal (n = 3), and thalamus (n = 1) for the transition period.

When a nondiagnostic biopsy was encountered, follow-up surgery (either stereotactic or open biopsy or tumor decompression) was recommended to nine patients, of whom two underwent three more stereotactic biopsies before a diagnosis could be made (lymphoma, n = 3; glioblastoma, n = 3; metastasis, n = 2; ependymoma, n = 1). These nondiagnostic biopsies were included in the analysis. Of the remaining four patients, two were successfully treated for radionecrosis and one for intracerebral abscess; the fourth was concluded to have an infarction after meticulous analysis for a demyelinating disease.

On average, 6.6 (range: 1 – 20) tissue samples were taken during each procedure with either frameless stereotaxy technique (Table 1). Table 4 shows the histological diagnoses that were made on the tissue samples taken by either system. In ten cases, seven in Medtronic Treon^TM^ (6.9 %) and three in BrainLAB® (2.9 %; p < 0.05), an intraoperative frozen-section diagnosis was requested. All of these included diagnostic biopsies.

**Table 4:** Histological diagnoses made on tissue samples acquired by stereotactic biopsy

	Stealth Treon^TM^(n = 102)	Transition period (n = 43)	BrainLAB® (n = 102)
Nondiagnostic	5	5	6
Malignant glioma	59	21	42
Low-grade glioma	9	5	15
Lymphoma	11	9	20
Metastasis	2	1	7
Infection/vasculitis/MS/abscess	12	1	9
Craniopharyngioma cysts^*^	2	1	1
Medulloblastoma	0	0	2
Others	2	0	0

MS = multiple sclerosis; others include malignant peripheral nerve sheath tumor and hematoma

## Discussion

The main purpose of stereotactical biopsy systems, frameless or otherwise, is to produce a safe, fast, reliable and easy diagnostic biopsy. For this purpose, many different systems are available to the neurosurgeon, each with its distinctive set of qualities. We present a retrospective comparison between the Medtronic Treon^TM^ Vertek® and the BrainLAB® Varioguide systems. Our diagnosis yield overall was 94.6 %. Morbidity and mortality profiles were also comparable with reports in the literature [1, 2, 7–10, 12, 16].

There was no difference in diagnostic yield between the two systems, which we defined as the likelihood of a biopsy producing pathological tissue to be analyzed, regardless of whether it could be included in the World Health Organization WHO/Central Nervous System CNS classifications [9], as there are still pathological entities that cannot be classified according to this system.

A descending trend can be noticed in the number of times the team asked for a frozen section histology examination to be performed during the operation, possibly reflecting the increase in experience and confidence using the frameless stereotactic systems. However, the operating time was significantly longer using the BrainLAB® Varioguide, in part probably pointing toward the learning curve, not pertaining to the act of biopsying, but to the system itself, as shown by a gradual decrease of 12.5 % in median operating time over the years. Nevertheless, in our cohort the total operating time remained approximately 11 %, or 10 min, longer when using the BrainLAB® system, even after correction for some well-established confounders.

Patients undergoing biopsy with the BrainLAB® Varioguide show significantly more frequent focal neurological signs postoperatively, but this was not correlated with increased hemorrhage or perilesional edema. Only speculations are possible as to why this has occurred, statistical evidence not pointing to any reason in particular. Some reports [18] suggest that the size of the needle correlates with the risk of hemorrhage, but this could not be established in our series (outer needle diameter 2.2 and 1.8 mm and cutting window length 7 and 10 mm for the Stealth Treon^TM^ Vertek® and Brainlab® Varioguide, respectively).

Two deaths occurred, one concerning an 81-year-old male with a parieto-occipital glioblastoma who suffered a basilar artery thrombosis 8 days after the operation. The second concerned a 53-year-old male with a multiloculated mixed anaplastic oligo-astrocytoma expanding the better part of the right cerebral hemisphere who suffered a large, deep intracerebral hematoma and subsequently expired 9 days after the operation. These cases are thus not attributable to the stereotactic systems themselves.

Using both systems we were able to reach a diagnostic yield that was higher even than those usually reported in the literature [2, 7, 9, 10, 15]. Counterintuitively, the intraoperative MRI-enhanced biopsies, which actively provide feedback as to the changes the lesion to be biopsied is undergoing, yielded the worst diagnostic yield [9].

We can thus conclude that both systems are effective, safe, and reliable tools, which when used properly have an excellent diagnostic yield, above 95 %. Despite minor differences, these tools are both part of a well-crafted diagnostic armamentarium available to contemporary neurosurgeons, which should be put to work in order to yield quick and precise diagnostic results. Future prospective studies are needed to compare the effectiveness of these and other available products, including parameters such as end-user satisfaction, subjective intraoperative stress, and overall comfort, among others.
